# Effect of an Interactive Training on Choosing Delivery Method among Primiparous Pregnant Women: An Interventional Study

**DOI:** 10.17533/udea.iee.v38n1e04

**Published:** 2020-02-26

**Authors:** Nahid Zarifsanaiey, Alireza Bagheri, Faezeh Jahanpour, Samaneh Nematollahi, Parviz Azodi

**Affiliations:** 1 Ph.D. Virtual School, Shiraz University of Medical Sciences, Shiraz, Iran. Email: sanaieyn@sums.ac.ir. Corresponding author. Shiraz University of Medical Sciences Shiraz Iran sanaieyn@sums.ac.ir; 2 M.Sc. Bushehr University of Medical Sciences, Bushehr, Iran. Email: bagheri54@yahoo.com Bushehr University of Medical Sciences Bushehr Iran bagheri54@yahoo.com; 3 Nurse, Ph.D. Nursing and Midwifery School, Bushehr University of Medical Sciences, Bushehr, Iran. Email: f_jahanpour@yahoo.com Bushehr University of Medical Sciences Bushehr Iran f_jahanpour@yahoo.com; 4 M.Sc. Shiraz University of Medical Sciences, Shiraz, Iran. Email: samane.nematolahi@yahoo.com Shiraz University of Medical Sciences Shiraz Iran samane.nematolahi@yahoo.com; 5 Nurse, M.Sc. Paramedical Faculty, Bushehr University of Medical Sciences, Bushehr, IR Iran. Email: azodi.parviz@gmail.com Bushehr University of Medical Sciences Bushehr Iran azodi.parviz@gmail.com

**Keywords:** pregnancy, parity, cesarean section, delivery, obstetric, unnecessary procedures., embarazo, paridad, cesárea, parto obstétrico, procedimientos innecesarios, gravidez, paridade, cesárea, parto obstétrico, procedimentos desnecessários.

## Abstract

**Objective.:**

To evaluate the effect of interactive training conducted during pregnancy on choosing delivery method among primiparous women.

**Methods.:**

Quasi-experimental study carried out in 2017 in two hospitals in the city of Bushehr (Iran), with the participation of 108 primiparous pregnant women in an educational program consisting of eight 2-hour sessions every two weeks in which interactive training activities were performed (group discussions, classroom sessions, and delivery of printed educational material) on themes related with physiological delivery, painless vaginal delivery methods, and complications of cesarean delivery without indication, among others. Before and after the intervention, the Knowledge and Preferred Method of Delivery Questionnaire by Moradabadi *et al*., was used to obtain information.

**Results.:**

The results indicated that the level of knowledge in the group of mothers increased significantly between the pre-intervention and post-intervention assessment (13.2 versus 19.4, of 20 possible maximum points; *p* <0.001). Additionally, significant difference was observed in the selection of the vaginal delivery method before and after the intervention (74.1% versus 98.1%; *p*<0.001).

**Conclusion.:**

Implementation of interactive training increased knowledge of pregnant women on the delivery and induced a positive effect to encourage the primiparous mothers to have a vaginal delivery.

## Resumen

**Objetivo.:**

Evaluar el efecto de un entrenamiento interactivo realizado durante el embarazo sobre la elección del método de parto en mujeres primíparas.

**Métodos.:**

Estudio cuasiexperimental realizado en 2017 en dos hospitales de la ciudad de Bushehr (Irán). 108 mujeres embarazadas primíparas participaron en un programa educativo consistente en ocho sesiones de dos horas de duración cada dos semanas, en las que se realizaron actividades de capacitación interactiva (discusiones grupales, clases magistrales y entrega de material educativo impreso) sobre temas relacionados con el parto fisiológico, los métodos de parto vaginal sin dolor, las complicaciones del parto por cesárea sin indicación, entre otros. Antes y después de la intervención se utilizó, para la toma de información, el *Knowledge and Preferred Method of Delivery Questionnaire* de Moradabadi *et al*.

**Resultados.:**

Los resultados indicaron que el nivel de conocimiento en el grupo de madres aumentó significativamente entre la evaluación preintervención a la posintervención (13.2 versus 19.4, de 20 puntos máximos posibles; *p*<0.001). Además, se observó una diferencia significativa en la selección del método de parto vaginal antes y después de la intervención (74.1% versus 98.1%; *p*<0.001).

**Conclusión.:**

La implementación de la capacitación interactiva aumentó el conocimiento de las embarazadas sobre el parto e indujo un efecto positivo para alentar a las madres primíparas a tener un parto vaginal.

## Resumo

**Objetivo.:**

Avaliar o efeito de um treinamento interativo realizado durante a gravidez na escolha do método de parto em mulheres primíparas.

**Métodos.:**

Estudo quase experimental realizado em 2017 em dois hospitais na cidade de Bushehr (Irã). 108 gestantes primíparas participaram de um programa educacional composto por oito sessões de duas horas a cada duas semanas, nas quais foram realizadas atividades de treinamento interativas (discussões em grupo, master classes e entrega de material educacional impresso) sobre questões relacionadas ao parto métodos fisiológicos, parto vaginal sem dor, complicações do parto cesáreo sem indicação, entre outros. Antes e após a intervenção, o Questionário de Conhecimento e Método Preferido de Entrega de Moradabadi et al.

**Resultados.:**

Os resultados indicaram que o nível de conhecimento no grupo de mães aumentou significativamente entre a avaliação pré-intervenção e a pós-intervenção (13.2 versus 19.4, de 20 possíveis pontos máximos; *p*<0.001). Além disso, foi observada diferença significativa na seleção do método de parto vaginal antes e após a intervenção (74.1% versus 98.1%; *p*<0.001).

**Conclusão.:**

A implementação do treinamento interativo aumentou o conhecimento das gestantes sobre o parto e induziu um efeito positivo para incentivar as primíparas a terem um parto vaginal.

## Introduction

Delivery is one of the most important and crucial services provided by the healthcare system in any community as every service should be provided with the minimum cost and mental-physical side effects.(1) The cesarean delivery method is no exception in this regard. It is necessary in cases where vaginal delivery is not safe for the mother and the child. The standard cesarean operation rate is 15% to 20% for the pregnant population in each society. The average rate of cesareans in Iran is reported at 40%, which is higher than the rate defined by the World Health Organization (5% to 15%). Studies show that 92% of these cesareans are without any urgent need and due to fear of pain and side effects of vaginal delivery.(2) In addition, it is higher than the cesarean rate in developed countries, such as the United States (33%) and England (32%).(3) 

According to the statistics issued by the Ministry of Health, Mothers' Health Department in 2011, out of 1.3-million registered births in the country, about 53% of deliveries were by cesarean section and 47% by vaginal methods, revealing high and disturbing cesarean section rates in the country.(4) In the Bushehr Province, the level of cesareans is higher than the standard, such that in 2013, 10,865 cases out of 20,500 deliveries (53%) were conducted through cesarean section. Indeed, the unreasonable increase in cesarean section delivery is one of the problems of health systems in all societies, and Iran is no exception to this rule. Certainly, many factors affect this situation. The rate of cesarean delivery has tripled compared to the 1970s, and the rate of increase is higher than expected. Increasing age of marriage, increasing age at the first delivery, increasing employment rate of women, and gaining access to health services, as well as advanced technology has placed them in a vicious cycle causing increased cesarean section deliveries.(5,6) 

Study results have suggested that the risk of maternal death from cesarean delivery is greater than that from vaginal delivery.(7) Other maternal cesarean risks include maternal morbidity during and after surgery, wound infections, infertility, and venous thrombosis of the feet. Cesarean delivery, if performed appropriately, helps reduce maternal mortality and morbidity.(8) Nowadays, definitive cesarean sectional indices, such as misalignment of the head with pelvis, placental or fetal mileage, peripheral placenta, premature pairing, umbilical cord prolapse, and severe preeclampsia are present. In all circumstances, the life of the mother or the fetus in the absence of intervention surgery is at risk, which is estimated to be between 5.8% and 8.8% for all birthdays.(6) 

Achieving the Millennium Development Goals (MDG) is one of the international obligations of Iran. One of the MDG indicators is maternal health. Cesarean delivery is one of the indicators used to monitor this goal.(9) Lack of awareness about the side effects of cesarean and negative views about vaginal delivery are among the most important causes of women’s tendency to cesarean method. Some researchers believe that the attitudes of doctors and midwives, as well as the degree of psychological support that women receive have a significant role for women in relation to the impact of giving birth. According to some researchers, the only way to reduce the rate of cesarean section is to inform and train women. Training should be provided for all patients, physicians, and nurses. Studies also show that women are influenced by external and internal factors, including doctors and midwives in decision-making for selecting the type of delivery.(10) Nowadays, education to the client is accepted as a part of the activities of all the health system staff with one of the important roles of midwives being their educational role. Effective teaching during pregnancy can play a significant role in reducing the number of illnesses and complications while also promoting health.(11) 

In this regard, guiding and teaching pregnant women can reduce unnecessary cesarean section cases where mothers can choose the appropriate method by gaining the required awareness and the physician's discretion, thereby, avoiding unnecessary cesarean cases. Carter *et al*., in their study aimed at examining the effect of pregnancy educational classes on maternal health. They found that classes during pregnancy could have a positive effect on the decision of mothers to choose a type of delivery.(12) Navaee, in a study, observed a significant reduction in the rate of cesarean sections in the trained group.(13) Darsareh *et al*.,(14) in a study conducted in 2016, pointed to the role of education in the form of a belief in the health model and its positive role in the tendency of pregnant women to choose vaginal delivery. In most review studies, education was investigated only via one educational method, especially lecture-based method while the use of other interactive strategies (lecture-based method and group discussion) has remained largely understudied. In accordance with the increasing importance of teaching pregnant women through interactive approaches, the present study aims to examine the effect of interactive educational intervention during pregnancy on the choice of delivery method at Bushehr University of Medical Sciences. 

## Methods

This research is an interventional study with a one group pre-test post-tests design, aimed at investigating the effect of organized interactive training during pregnancy on the choice of delivery method in primigravidae women referring to prenatal clinics of two hospitals located in Bushehr city, Iran.

Samples and setting. A convenience sample included 130 pregnant women referred to prenatal clinics of two hospitals in Bushehr, Iran. The study was conducted between November 2017 and June 2018. Eligible subjects were primigravidae women aged 18 to 45 years; they were at most at 28 weeks of gestation and were willing to participate in the research while living in Bushehr city. Exclusion criteria involved any medical or pregnancy complications during the intervention and elective cesarean section, unwillingness to continue collaboration in the research, and not attending the training sessions during the study. Up to implementation of the study, 22 of women did not attend more than one session due to travel and family problems; so they were excluded from the study and 108 women were analyzed (*n*=108).

Intervention. After receiving approval from the Vice Chancellor for Research and Ethics Committee of the University, required arrangements were made with the Educational administration of Bushehr University of Medical Sciences. Then, the researchers started to develop appropriate contents related to maternity care through library browsing as well as valid national and international papers. The instructional content included an introduction to routine pregnancy training, physiological delivery, and various vaginal delivery methods without pain, complications of cesarean delivery without indication, the facilities of labor delivery room and the maternity ward to assure the mother, practical exercises including stretching, breathing, and relaxing exercises. The instructional content was approved by five faculty members of the Department of Maternity Nursing at Nursing and Midwifery School of Shiraz University of Medical Sciences.

After presenting the research setting, the sampling was made and the study objectives were explained. The written informed consent was obtained from the women. Initially, the levels of knowledge and selected method of delivery were examined in all participants before the training. Then, the research samples underwent eight 2-hour sessions of organized interactive training classes within 16 weeks (every two weeks). One week following the last intervention session, the levels of knowledge and selected method of delivery of the research samples were assessed again. In each 2-hour session (eight sessions), a short lecture was taught by one of the researchers qualified in this area. Thereafter, in addition to modifying the learning environment and seating arrangements, the women engaged in 10 small groups to discuss the topic and share their experience, as well as challenges with each other. The professor played the role of facilitator at this stage, provided the necessary guidance to the research samples, and encouraged them to participate in the discussion. In the next stage, the instructor provided additional information and explained the educational content using PowerPoint and instructional videos, while also answering questions from the participants. During each class, interaction among participants, as well as between participants and teachers was encouraged. To supplement the presentation and provide a more effective program, at the end of each session, a pamphlet was given to the mothers to review the content at home.

Data collection tools. a) *Demographic information* (age, age of marriage and pregnancy, level of education, and occupation of the participants and their spouses); and b) *Knowledge and Preferred Method of Delivery* questionnaire: this questionnaire was developed by Moradabadi *et al.,*(15) to measure knowledge of the advantages and disadvantages of various delivery methods and selected method of delivery. The study by Moradabadi *et al.*,(15) content validity was used to approve the validity of the questionnaire where it was given to five experts (two healthcare specialists, two gynecologists, and a statistician). Test-retest was used to evaluate the reliability of the research instrument. A reliability coefficient of 75% was estimated and approved. This tool contains 20 multiple-choice questions, scored from 0 to 1. The total score of the respondents ranges from 0 to 20.

Ethical issues. At the beginning of the training program, after introducing himself, the researcher explained to the pregnant women the aims of the study and the written consent was obtained from all the participants. Also, participants were assured that all information collected from them would remain confidential. 

Statistical Analysis. Data analyses were performed by using SPSS version 16; results of the analyses were provided in form of descriptive and inferential data. A significance level of 0.05 was considered for the tests.

## Results

In this study, 108 pregnant women completed the research process. The mean age of the participants in the study was 27.7 years, ranging between 16 and 37 years, while the marriage age varied from 14 to 33 years. The most frequent levels of education among the study participants and their spouses were diplomas and college degree with frequencies of 38.9% and 44.4%, respectively.

The first objective in this research was to compare the participants' preferred method of delivery before and after the intervention. As can be seen in Table 1, the McNemat test showed that most pregnant women had a greater tendency to vaginal delivery after the intervention (*p*<0.001). 

**Table 1:** Comparison of the participants’ Preferred Method of Delivery before and after the intervention

Selected delivery method	Sub-Group	n	%
Before the intervention	Cesarean	28	25.9
Before the intervention	Vaginal	80	74.1
After the intervention	Cesarean	2	1.9
After the intervention	Vaginal	106	98.1

The second objective of this study was to compare the participants’ knowledge about the method of delivery before and after the intervention. As can be observed in the Table 2, the Wilcoxon test revealed significant difference between before and after the intervention on the level of knowledge of pregnant mothers (*p*<0.001).

**Table 2:** Comparison of the Mean ± SD of knowledge before and after educational intervention in the pregnant women

		Mean ± SD	Min.	Max.
Knowledge	Before the intervention	13.2±4.0	3	20
Knowledge	After the intervention	19.4±1.0	4	20

The sociodemographic variables in this study did not act as confounders in the relationship between the delivery method selection and having or not having received the intervention.

## Discussion

The present study was conducted to investigate the effect of an organized Interactive approach (group discussion, lecture presentation with questions and answers plus educational pamphlets) on knowledge and preferred method of delivery in pregnant women. The results revealed a significant increase in the pregnant women's level of knowledge, and they displayed a positive attitude toward vaginal delivery after the intervention. Our study also indicated that Interactive education leads to improved level of knowledge in the pregnant women. In an interactive learning environment, a variety of individual and group learning techniques should be used to enhance learning. In this regard, one of the interactive educational methods in our research was group discussion. Working within a group has greater advantages for learners, as they share their views, observations, and previous experiences with each other and create new knowledge.(18,19) 

Furthermore, individual learning is a very important element in the interactive learning environment. Individual learning emphasizes that learning can take place anytime, anywhere with any speed. We used the educational pamphlet to promote learning independently in our research. The pregnant women in our study were given a pamphlet at the end of every session. This method allowed them to interact with educational content and to learn at their desired time, location, and learning pace.(17) This finding is in line with similar results regarding the effects of an educational pamphlet on knowledge and anxiety in women with preeclampsia,(20) on pregnant women with fear of pain of childbirth,(21) and on the level of women’s knowledge. The results revealed that the levels of women’s knowledge were improved after the intervention. However, unlike the results obtained from the present study, other studies, such as those by Kjaergaard *et al*.,(22) and Kosan *et al.,*(23) no significant difference was observed between education and level of pregnant women’s knowledge and selecting the vaginal delivery method. The difference in the results may be due to the training methods used in those studies and the underlying variables of the study samples.

The findings of the present research also showed a significant relationship between age and height and selected delivery method. The younger and shorter women had a greater tendency to selecting cesarean method. This can be due to social factors such as the importance of beauty and the prevention of body deformation, changes in lifestyle, intolerance to labor pain, and fear of pain, followed by worry about fetal health, and fear of reproductive system rupture during vaginal delivery.(21,22,24) Interestingly, there was no difference after the intervention. 

In conclusion, the findings of the study showed that training pregnant women and designing interactive programs to educate them could have a significant role in increasing their knowledge and reducing the rate of selecting cesarean section without indication. Thus, to prevent the side effects of cesareans in mothers and avoid the higher care costs, it is suggested to use various ways to inform mothers about routine pregnancy training, physiological delivery, and various pain-free vaginal delivery methods, complications of cesarean delivery without indication, the facilities of labor delivery room and the maternity ward to assure the mother, as well as practical exercises including stretching, breathing, and relaxing exercises.

Limitations: This study was performed in only two clinics and needs to be implemented on a wider scale for generalization. Also, only short-term effects were studied, there is a need for long-term follow-ups.

## References

[1] Tunçalp Ӧ, Were WM, MacLennan C, Oladapo OT, Gülmezoglu AM, Bahl R (2015). Quality of care for pregnant women and newborns-the WHO vision. BJOG.

[2] O'donovan C, O'donovan J (2018). Why do women request an elective cesarean delivery for non‐medical reasons? A systematic review of the qualitative literature. Birth.

[3] Kim SJ, Han KT, Kim SJ, Park EC, Park HK (2016). Impact of a diagnosis-related group payment system on cesarean section in Korea. Health Policy..

[4] Banaei M, Pormehr-Yabandeh A, Roozbeh N (2018). Evaluation of the cesarean section rate and its reasons in Shariati Hospital of Bandar Abbas in 2015.. J. Adv. Pharm. Edu. Res.

[5] Yousefzadeh S, Esmaeili DM, Asadi YM, Shakeri MT. (2016). Effects of Training about the Benefits of Natural Childbirth during Pregnancy on the Attitude and Intentions to Select the Mode of Delivery in Nulliparous Women. J. Midwifery Reprod. Health..

[6] Magne F, Puchi Silva A, Carvajal B, Gotteland M. (2017). The elevated rate of cesarean section and its contribution to non-communicable chronic diseases in Latin America: the growing involvement of the microbiota. Front. Pediatr..

[7] Molina G, Weiser TG, Lipsitz SR, Esquivel MM, Uribe-Leitz T, Azad T (2015). Relationship between cesarean delivery rate and maternal and neonatal mortality. JAMA.

[8] Piroozi B, Moradi G, Esmail N, Ghasri H, Farshadi S, Farhadifar F. (2016). Evaluating the effect of health sector evolution plan on cesarean rate and the average costs paid by mothers: A case study in Kurdistan province between 2013-2015. J. Hayat..

[9] Moghadam ZB, Khiaban MO, Esmaeili M, Salsali M. (2018). A Review of the High Level of Education and Reduced Fertility in Iranian Women: Have Women Been Empowered?. Int. J. Womens Health Reprod. Sci..

[10] Ulloa IM, Muñoz L. (2019). Care from the cultural perspective in women with physiological pregnancy: a meta-ethnography.. Invest. Educ. Enferm..

[11] Osorio-Castaño JH, Carvajal-Carrascal G, Rodríguez-Gázquez M. (2017). Preparation for motherhood during Pregnancy: a Concept Analysis.. Invest. Educ. Enferm..

[12] Carter EB, Temming LA, Akin J, Fowler S, Macones GA, Colditz GA, Tuuli MG. (2016). Group prenatal care compared with traditional prenatal care: a systematic review and meta-analysis. Obstet. Gynecol.

[13] Navaee M, Abedian Z. (2015). Effect of role play education on primiparous women's fear of natural delivery and their decision on the mode of delivery.. Iran. J. Nurs. Midwifery Res.

[14] Darsareh F, Aghamolaei T, Rajaei M, Madani A, Zare S (2016). The differences between pregnant women who request elective caesarean and those who plan for vaginal birth based on Health Belief Model. Women. Birth.

[15] Safari-Moradabadi A, Mehraban M, Alavi A, Pormehr-Yabandeh A, Ghiaspour T, Dadipoor S (2018). Investigating the delivery type among primiparous women in Bandar Abbas according to the health belief model. World fam. Med..

[16] Laluei A, Kashanizadeh N, Teymouri M. (2009). The influence of academic educations on choosing preferable delivery method in obstetrics medical team: investigating their viewpoints.. Iran. J. Med. Educ..

[17] Quick KK, Blue CM. (2019). Using Situated Learning Theory to Build an Interactive Learning Environment to Foster Dental Students’ Professionalism: An Ignite Project.. J. Dent. Educ..

[18] T O. Nyumba, Wilson K, Derrick CJ, Mukherjee N (2018). The use of focus group discussion methodology: Insights from two decades of application in conservation. Methods Ecol. Evol..

[19] Cox SN, Guidera KE, Simon MJ, Nonyane BA, Brieger W, Bornman MS, Kruger PS (2018). Interactive malaria education intervention and its effect on community participant knowledge: the malaria awareness program in Vhembe district, Limpopo, South Africa.. Int. Q. Community Health Educ..

[20] Gingras-Charland ME, Côté AM, Girard P, Grenier A, Pasquier JC, Sauvé N (2019). Pre-eclampsia Educational Tool Impact on Knowledge, Anxiety, and Satisfaction in Pregnant Women: A Randomized Trial.. J. Obstet. Gynecol.

[21] Kızılırmak A, Başer M. (2016). The effect of education given to primigravida women on fear of childbirth.. Appl. Nurs. Res..

[22] Kjærgaard H, Wijma K, Dykes AK, Alehagen S (2008). Fear of childbirth in obstetrically low‐risk nulliparous women in Sweden and Denmark. J. Reprod. Infant Psychol..

[23] Kosan Z, Kavuncuoglu D, Calıkoglu EO, Aras A. (2019). Delivery preferences of pregnant women: Do not underestimate the effect of friends and relatives.. J Gynecol. Obstet. Hum. Reprod..

[24] Sotoodeh Jahromi A, Rahmanian K, Madani A. (2018). Relation of Knowledge about Cesarean Disadvantages and Delivery Mode Selection in Women with First Pregnancy; South of Iran.. J. Res. Med. Dent. Sci..

